# Fourth International Congress on School Hygiene

**Published:** 1913-06

**Authors:** 


### Fourth International Congress on School Hygiene.

The fourth International Congress on School Hygiene will be held in Buffalo, New York, August 25-30, 1913, under the patronage of Mr. Woodrow Wilson, President of the United States. President—Charles W. Eliot, President Emeritus, Harvard University.

Vice-Presidents—Dr. William H. Welch, Professor of Pathology, Johns Hopkins University; Dr. Henry P. Walcott, Chairman Massachusetts Board of Health.

ANNOUNCEMENT OF THE PROGRAM COMMITTEE.

I. The program committee is particularly anxious that the papers presented at this congress shall deal largely with results secured through the practical application of scientific facts and procedures of school hygiene, and with the results of scientific investigation and laboratory research. Teachers, investigators, physicians, dentists, sanitarians and public-spirited citizens who have evidence showing that the school hygiene under their observation has been effective and of real service, or who can present scientific facts proven by their labors, will be welcome contributors.

II. The committee is further anxious to secure papers relating to rural school hygiene and village school hygiene as well as to city school hygiene. The problems of the city schools have received a great deal of much deserved attention. The very serious problems of the village school and the rural school have received but little attention. The study and the solution of these problems are of obvious complexity and importance.

III. The following rules have been established by the program committee:

1. The official languages of this congress shall be English, French, German, Spanish and Italian.

2. Papers must be limited to fifteen minutes in presentation.

3. Manuscripts submitted for publication in the proceedings must not exceed 3000 words.

4. Time will be assigned on each program for the open discussion of groups of related papers.

5. Individuals participating in the open discussion may be limited to three minutes each, and such discussion may be terminated at the discretion of the presiding officer. This rule is laid down for the purpose of enabling the presiding officer to put his schedule through on time. Preliminary abstracts will not be published.

6. Authors are requested to send in their manuscripts early. All such manuscripts must be in the hands of the secretary general by July 1, 1913.

7. The program committee reserves the right to reject any contribution which for any reason it feels should not be accepted.

8. Papers will not be read in absentia, but such papers may be published in the proceedings.

9. Exceptions to these rules may be made only with permission from the program committee.

IV. The program will be organized into sections, as follows. These sections will be subdivided whenever such division appears to be desirable.

Section 1. "The Hygiene of School Buildings, Grounds, Material Equipment and Upkeep." This section will include papers on topics related to the location, plan, construction, equipment and upkeep of city, village and rural schools, open-air schools, private schools, boarding schools, summer camps and special schools for backward, truant, delinquent, deficient, defective and deformed children—i. e., site, architecture, decoration, ventilation, illumination, cleaning system, plumbing, toilets, sewage disposal, school furniture, school books, water supply, drinking facilities, bathing facilities, swimming pools, school grounds, school athletic fields, fields for games, sport and play, lunch rooms and equipment, gymnasium, social rooms, rest rooms, libraries, laboratories, class rooms, study rooms and lecture rooms.

Section 2. "The Hygiene of School Administration, Curriculum and Schedule." This section will include all topics concerned with the hygienic factors found in school administration, curriculum and schedule as they apply to country, village and city schools; and to the modifications necessary for the best interest of our various special schools. Papers on such subjects as the following would belong to this section: Hygiene of the teacher; hygiene of the child; hygiene of the janitor and other school employes; hygiene of the schedule, growth and age; school fatigue; need for and management of school lunches and school baths; influence of the seasons; study periods; home work; recesses; vacations; athletics; the problems of heredity in relation to school hygiene; overcrowding; the teaching of hygiene; the training of teachers of hygiene; special phases of hygiene: as personal hygiene, oral hygiene; preventive hygiene; educational hygiene; community hygiene; sex hygiene; play; physical education; domestic hygiene; puericulture, and first aid; special plans for and results from the instruction of backward children, truant, delinquent and crippled children; the economics of school hygiene; relation to the home.

Section 3. "Medical, Hygienic and Sanitary Supervision in Schools." This section will receive papers on the management, operation and results of medical, hygienic and sanitary supervision in public, private and special, country, village and city schools, colleges, universities and professional schools.

Such subjects as the following will be included: The control of health inspection; sanitary supervision; the organization of health departments in schools; the relationship to the board of health; the equipment, training and compensation of school physicians; school nurses; school clinics; relation to health supervision in the schools to the practice of the physician, the dentist, and the hospital; relation of medical and hygienic supervision in the schools to health supervision in the home; standardization of examinations; sanitary supervision of school rooms (class rooms), locker rooms, swimming pools, toilets, school books and school furniture; supervision of disease carriers; prevention of epidemics; follow up methods and results; medical inspection and treatment; standardization of records.

---

Removal Notice.—A research laboratory has recently been established in New York city, at No. 428 Lafayette street, near Astor Place, with Dr. Fenton B. Turck, formerly of Chicago, as director, to continue his work on the various problems connected with the alimentary tract, under an endowment by two former patients of London, England.

Dr. Turck has removed his office and residence from Chicago to Fourteen East Fifty-third street, New York, there devoting his morning hours to office practice, the later hours of the day being taken by the research work at the laboratory.

The director is at present assisted by the following staff:

Organic Chemistry—A. R. Rose, formerly of University of Minnesota and Yale; Arthur Knutson, formerly of University of Missouri and Columbia; Katherine R. Coleman, formerly of Columbia University.

Physiological Chemistry—Vincent Greco, University of Palermo, Italy.

Bacteriology—Otto Maurer, K. Oberrealschule, Heilbroun, Germany, and University of Wisconsin; W. W. Browne, formerly of Brown University.

General Pathology—P. J. Friedman, formerly of department of health, City of New York Research Laboratory, and Earle Kister, University of Toronto, Canada.

---

Only to Purify the Blood.—"Hab yo' any medicine dat will purify de blood?"

"Yes, we keep sarsaparilla, at one dollar a bottle. It purifies the blood and cleaers the complexion."

"Well, boss, ain't yo' got sumfin' fo' about fifty cents, jess fo' de blood? I don't keer about de complexion."

---

Diagnosis.—"Sure and you had the doctor to see your husband, did ye?"

"Yes! He gave him a bottle o' bark and pupsin and a canine pill."

"Begorra! It's hydrophobia he has, I guess!"—*Medical Council.*

---

A Sure Remedy.—"Did you ever call upon Dr. Banquet, professionally?"

"Yes, once. I was drowning."

"Drowning?"

"Yes. He diagnosticated my case on the instant and wrote a prescription on a chip, which he threw into the water where I could get it."

"What was the prescription?"

"℞: Swim."

Unless a foreign body has been definitely localized at some distance from the point of entry, this, when known, should be included in the incision for removal, marking this spot for further reference by a little nick in the skin.—*American Journal of Surgery.*

---

In the digits the dissection for a foreign body should be confined, if possible, to a quadrant bounded by the tendons in the median line and the vessels and nerve on each side.—*American Journal of Surgery.*

